# Diagnostic Performance of Clinical Laboratory Indicators With Sarcopenia: Results From the West China Health and Aging Trend Study

**DOI:** 10.3389/fendo.2021.785045

**Published:** 2021-12-10

**Authors:** Mengting Yin, He Zhang, Qianhui Liu, Fei Ding, Yiping Deng, Lisha Hou, Hui Wang, Jirong Yue, Yong He

**Affiliations:** ^1^ Department of Laboratory Medicine, West China Hospital, Sichuan University, Chengdu, China; ^2^ Department of Geriatrics and National Clinical Research Center for Geriatrics, West China Hospital, Sichuan University, Chengdu, China

**Keywords:** sarcopenia, AST/ALT ratio, fasting insulin, diagnostic performance, metabolism

## Abstract

**Background:**

Sarcopenia is an age-related and skeletal muscle disorder involving the loss of muscle mass or strength, and physiological function. Although the diagnostic indicators used in the different guidelines are for muscle mass, strength and physical performance, there are currently no uniform diagnostic criteria. Therefore, we aimed to explore the relationship between a series of biomarkers with sarcopenia in southwest China.

**Methods:**

We included 4302 patients from West China Health and Aging Trend (WCHAT) study. Sarcopenia was defined according to the Asian Working Group for Sarcopenia: 2019 Consensus Update on Sarcopenia Diagnosis and Treatment. Thyroxine、albumin、total protein、prealbumin、albumin to globulin ratio (A/G)、25(OH)VD、fasting insulin、adrenal cortisol、triglyceride、high-density lipoprotein、hemoglobin and aspartate aminotransferase to alanine aminotransferase ratio (AST/ALT) were measured. The receiver operating characteristic curves (ROC) were established to describe the predictive value for sarcopenia and we also used multivariate logistic regression analysis to identify risk factors of the disease.

**Results:**

In terms of protein state, patients with sarcopenia had lower value in total protein, albumin, prealbumin, A/G than the control (*P*<0.001). Patients had lower value in triglyceride but higher value in high-density lipoprotein compared with the healthy in the indicators of lipid metabolism (*P*<0.001). In the aspect of hormone state, patients had lower free triiodothyronine, fasting insulin but higher free tetraiodothyronine and adrenal cortisol than the healthy (*P*<0.001). The fasting insulin level (AUC=0.686) and the AST/ALT ratio (AUC=0.682) were the best predictors of sarcopenia among biomarkers. The diagnostic performance of fasting insulin combined with the AST/ALT ratio (AUC=0.720) is equal to multiple indicators (AUC=0.742).

**Conclusion:**

The fasting insulin combined with the AST/ALT ratio exhibits good diagnostic performance for sarcopenia.

## Introduction

Sarcopenia is an age-related and skeletal muscle disorder involving the loss of muscle mass or strength and physiological function (1). It`s reported that the prevalence of sarcopenia according to the Asian Working Group for Sarcopenia (AWGS) 2014 criteria ranged from 5.5% to 25.7% (2, 3). With the aggravation of the aging population in our country and the increased adverse health outcomes associated with sarcopenia including frailty, falls, disability and mortality, it`s necessary for the clinic to make an early diagnosis and intervention. In addition to clarifying the relationship between aging and sarcopenia, nutrition, endocrine dysfunction, glucose, and lipid metabolism as well as metabolic syndrome (Mets) have received more and more attention. The pathophysiology of sarcopenia may be that 1) a decline in muscle fiber numbers with aging and an increase in fibrosis leads to muscle dysfunction; 2) the imbalance between muscle protein synthesis and regeneration leads to loss of skeletal muscle; 3) on the one hand, insulin resistance leads to the dysfunction of the use of glucose, on the other hand, it leads to muscle atrophy (4–6). Now, the diagnosis of sarcopenia requires objective measurements of muscle mass, muscle strength, and physical performance. Currently, dual-energy X-ray absorptiometry (DXA), and bioelectrical impedance analysis (BIA) were the most commonly used in Asia for appendicular skeletal muscle mass measurement. Handgrip strength was widely recommended to indicate skeletal muscle strength (1, 7, 8). Although the diagnostic indicators used in the different guidelines are for muscle mass, strength, and physical performance, there are currently no uniform diagnostic criteria and the results measured by different devices cannot be directly compared. Despite promising advances in evaluating muscle mass and strength, it`s hard to implement in some primary hospitals in China due to the lack of equipment. The physical performance including Short Physical Performance Battery (SPPB), 6-min walk test, and the stair climb power test (SCPT) were not available for some elderly patients either. Meanwhile, the mechanism of sarcopenia has not been fully characterized. Therefore, we used the data from the West China Health and Aging Trend (WCHAT) study to explore the relationship between clinical laboratory biomarkers with sarcopenia.

## Methods

The current research is a cross-sectional analysis including baseline data of WCHAT study which was approved by the Ethical Review Committee (reference: 2017-445). The method of sampling is multi-stage cluster sampling and the response rate was 50.2% in the baseline data collection. And this research is supported by Grant No.2020YFC2005600/03 from the National Key R&D Program of China.

### Study Participants

Date selected from baseline of the WCHAT study, which was initiated in 2018 and included 7536 people aged 50 or older in the west China, were used in this analysis (9, 10). The exclusion criteria for them were as follows: 1) cognitive impairment; 2) a recent history of malignancy; 3) missing data on the laboratory measurements; 4) Diabetes Mellitus. Finally, a total of 4120 elderly patients from various communities in Sichuan Province, southwest of China, were admitted into this study, 786 of whom were diagnosed with sarcopenia. All participants were willing to take part in this study and informed consent was signed. The study was conducted in accordance with the Declaration of Helsinki and was approved by the Ethics Committee of Sichuan University.

### Sarcopenia Assessment

We used the AWGS 2019 as the diagnostic criteria, which was widely exerted in the diagnosis of sarcopenia in Asia, considering both the loss in muscle mass, muscle strength as well as physical performance. According to the AWGS, appendicular muscle mass (male: <7.0kg/m^2^, female: <5.7 kg/m^2^) for BIA, is considered as a loss of muscle mass. The AWGS also suggests that handgrip strength of <28kg and <18kg for men and women is defined as low muscle strength. The 6 m walking test <1.0 m/s and SPPB ≤9 are recommended for the evaluation of physical ability. Patients are definitely diagnosed with sarcopenia when low muscle strength and poor muscle function are confirmed. Meanwhile, if low physical performance is also accompanied sarcopenia is considered to sever.

### Specimen Collection

We collected specimen from the antecubital vein after an overnight fast. Biomarkers including fasting insulin, 25(OH)VD, alanine aminotransferase (ALT), aspartate aminotransferase (AST), total protein (TP), prealbumin (PA), albumin (ALB), free triiodothyronine (FT3), free tetraiodothyronine (FT4), triglycerides (TG), high-density lipoprotein (HDL), very low-density lipoprotein (VLDL), plasm total cortisol (PTC) and hemoglobin (Hb) were measured. Except Hb was used with whole blood, the remaining blood was centrifuged at 3000rpm for 10 minutes to obtain serum. TP, ALB, FT3, FT4, TG, VLDL, HDL, PTC were measured by electrochemiluminescence (cobas e801, Roche). ALT and AST were measured by enzyme method (cobas e801, Roche). PA was measured by Turbidimetric inhibition immuno assay (IMMAGE 800, Beckman). Some clinic signs such as weight, height, calf circumference (CC), waistline, mid-arm circumference (MAC), thickness of triceps skinfold (TST), hip circumference (HIPL) were also measured. Trained interviewers collected questionnaire data through face-to-face, one-on-one personal interviews. Trained technicians performed the anthropometric and bioimpedance measurements.

### Statistical Analysis

Kolmogorov-Smirnov test was used to confirm the normal distribution. Measurement data on normal distribution were expressed as mean ± standard deviation (M ± SD), and tested by an independent t-test. Abnormal distribution data were expressed as the median (interquartile range, IQR) and tested by the Mann-Whitney U test. Multivariate logistic regression was performed, including all univariately associated variables (*P* < 0.05). The analyses above were completed *via* SPSS software 25.0 (Chicago, IL, USA). All *P*-values were two-tailed and statistical significance was set at *P* < 0.05.

## Results

### Characteristics of the Study Cohorts

Clinical characteristics of the cohorts are described in (**Table 1**) and (**Supplementary Table 1**). Patients with sarcopenia had lower value in height, weight, waistline, HIPL, CC, MAC and TST than the healthy (*P*<0.001). In terms of nutrition state, patients with sarcopenia had lower value in TP, ALB, and PA than the control (*P*<0.001). Meanwhile, the level of A/G ratio in the sarcopenia group was lower than in the control (*P*<0.001). Patients had lower value in TG and VLDL but higher value in HDL compared with the healthy in the indicators of lipid metabolism (*P*<0.001). In the aspect of hormone state, patients had lower FT3, fasting insulin but higher FT4 and PTC than the healthy (*P*<0.001).

**Table 1:** Patient demographics.

	Sarcopenia (n = 810)	Control (n = 3400)	*P* value
**gender[n(%)]**			0.000
Male	328 (41.7)	1129 (33.9)	
Female	482 (58.3)	2271 (66.1)	
Age(years)	67.98 ± 8.66	61.03 ± 7.55	0.000
FT3/(pmol/L)	4.40 ± 0.80	4.61 ± 1.27	0.000
FT4/(pmol/L)	18.29 ± 2.94	17.88 ± 3.12	0.003
Fasting insulin	6.00 ± 4.96	8.97 ± 10.06	0.000
TP/(g/L)	71.17 ± 5.33	72.00 ± 5.95	0.000
ALB/(g/L)	43.24 ± 3.39	44.39 ± 2.96	0.000
PA/(g/mL)	262.20 ± 108.00	289.60 ± 160.40	0.000
A/G	1.58 ± 0.25	1.65 ± 0.26	0.000
TG(mmo/L)	1.60 ± 1.45	1.90 ± 1.81	0.000
VLDL(mmol/L)	0.73 ± 0.66	0.86 ± 0.82	0.000
HDL(mmo/L)	1.36 ± 0.35	1.26 ± 0.30	0.000
AST(U/L)	29.8 ± 17.8	29.2 ± 13.4	0.000
ALT(U/L)	23.1 ± 14.0	28.7 ± 19.7	0.000
25(OH)VD/(nmol/L)	18.34 ± 6.45	19.19 ± 6.25	0.001
AST/ALT	1.44 ± 0.50	1.16 ± 0.39	0.000
Hb/(g/L)	146.30 ± 17.59	149.10 ± 17.47	0.000
PTC(nmol/L)	372.90 ± 174.70	340.30 ± 137.90	0.000
Height/cm	152.71 ± 8.11	157.15 ± 7.93	0.000
Weight/kg	51.84 ± 8.91	64.40 ± 10.51	0.000
Waistline/cm	79.44 ± 9.12	89.08 ± 10.41	0.000
HIPL/cm	89.39 ± 6.10	96.75 ± 7.22	0.000
CC/cm	31.73 ± 2.75	35.51 ± 2.98	<0.001
MAC/cm	25.74 ± 3.00	29.59 ± 3.02	<0.001
TST/cm	19.87 ± 7.52	25.12 ± 8.37	<0.001

FT3, free triiodothyronine; FT4, free tetraiodothyronine; TP, total protein; ALB, albumin; PA, prealbumin; TG, triglyceride; AST/ALT, aspartate aminotransferase to alanine aminotransferase ratio; A/G, albumin to globulin ratio; HDL, high-density lipoprotein; LDL, low-density lipoprotein; PTC, adrenal cortisol; TST, triceps skinfold thickness; MAC, mid-arm circumference; CC, calf circumference.

### Risk Factors for Sarcopenia

Based on the findings of the univariate analysis, the following parameters were entered in the multivariate logistic regression model: fasting insulin, AST/ALT ratio, TP, PA, ALB, A/G, FT3, FT4, TG, HDL, HGB PTC,25(OH)VD. After the adjustment, the odds ratios (ORs) of sarcopenia showed that fasting insulin (OR=0.904, [95%CI: 0.882-0.927] *P*=0.000), AST/ALT ratio (OR=1.920, [95%CI: 1.564-2.358] *P*=0.000), HDL (OR=1.917, [95%CI: 1.413-2.600] *P*=0.000), TG (OR=1.109, [95%CI: 1.007-1.220] *P*=0.035), PA (OR=0.998, [95%CI: 0.997-1.000] *P*=0.022), FT4 (OR=1.084, [95%CI: 1.047-1.122] *P*=0.000), PTC (OR=1.001, [95%CI: 1.000-1.002] *P*=0.005) and 25(OH)VD (OR=0.980, [95%CI: 0.966-0.994] *P*=0.006) were identified as risk factors for the sarcopenia (**Table 2**).

**Table 2:** Association between biochemical indicators and sarcopenia according to unadjusted and adjusted logistic regression models.

	Unadjusted OR	95%CI	*P* value	Adjusted OR	95%CI	*P* value
Fasting insulin	0.867	0.847-0.887	0.000^***^	0.904	0.882-0.927	0.000^***^
PA	0.995	0.994-0.997	0.000^***^	0.998	0.997-1.000	0.022^*^
ALB	0.882	0.869-0.906	0.000^***^	0.971	0.895-1.054	0.478
AST/ALT	3.86	3.244-4.592	0.000^***^	1.920	1.564-2.358	0.000^***^
HDL	2.724	2.145-3.461	0.000^***^	1.917	1.413-2.600	0.000^***^
FT3	0.714	0.635-0.803	0.000^***^	0.894	0.795-1.005	0.060
FT4	1.036	1.011-1.062	0.004^***^	1.084	1.047-1.122	0.000^***^
TG	0.851	0.795-0.910	0.000^***^	1.109	1.007-1.220	0.035^*^
A/G	0.397	0.292-0.540	0.000^***^	0.521	0.213-1.277	0.154
PTC	1.001	1.001-1.002	0.000^***^	1.001	1.000-1.002	0.005^**^
TP	0.967	0.951-0.983	0.000^***^	1.017	0.971-1.066	0.465
HGB	0.99	0.985-0.994	0.000^***^	0.999	0.993-1.005	0.683
25(OH)VD	0.979	0.966-0.001	0.001^***^	0.980	0.966-0.994	0.006^**^

Model was adjusted for age and sex. ^*^P < 0.05, ^**^P < 0.01, ^***^P < 0.001. PA, prealbumin; ALB, albumin; AST/ALT, aspartate aminotransferase to alanine aminotransferase ratio; HDL, high-density lipoprotein; FT3, free triiodothyronine; FT4, free tetraiodothyronine; TG, triglyceride; A/G, albumin to globulin ratio; PTC, adrenal cortisol; TP, total protein; HGB, hemoglobin; 25(OH)VD, 25 hydroxyvitamin D.

### The Predictive Value of Indicators for Sarcopenia

**Figure 1:**
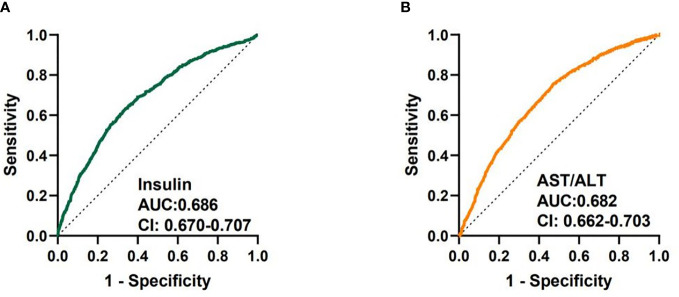
Receiver-operating characteristic curves (ROC) used for sarcopenia of fasting insulin and AST/ALT ratio. **(A)** ROC curve analysis of fasting insulin together with AUC and 95% confidence interval values. **(B)** ROC curve analysis of AST/ALT ratio together with AUC and 95% confidence interval values.

**Figure 2:**
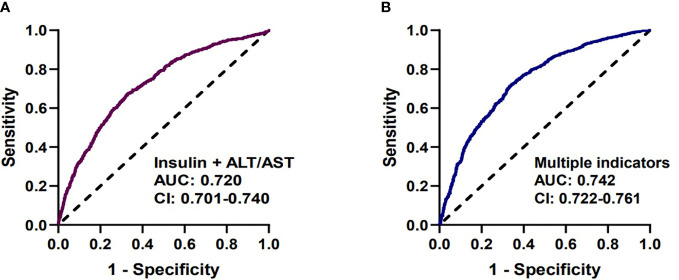
Receiver-operating characteristic curves (ROC) used for sarcopenia. **(A)** The predictive performance of insulin and AST/ALT (AUC=0.720 [95%CI: 0.701-0.740]). **(B)** The predictive performance of multiple factors, including fasting insulin, AST/ALT ratio, PA, HDL, FT4, PTC, 25(OH)VD (AUC=0.742 [95%CI: 0.722-0.761]).

We analyzed the diagnostic performance of indicators above through the receiver-operating characteristic curves (ROC). We found that fasting insulin AUC = 0.686 [95%CI: 0.670-0.707] and AST/ALT ratio AUC = 0.682 [95%CI: 0.662-0.703] had better predictive value than other parameters (**Figure 1**). The diagnostic efficacy of FT3 AUC = 0.574 [95%CI: 0.552-0.597] was significantly higher than that of FT4 AUC = 0.548 [95%CI: 0.525-0.570], and the difference was statistically significant *P* < 0.001. The index reflecting the protein levels, the diagnostic efficiency of sarcopenia from large to small was PA > ALB > A/G> TP, and the corresponding AUC were 0.608 [95%CI: 0.586-0.630], 0.604 [95%CI: 0.581-0.626)], 0.564 [95%CI: 0.542-0.586], 0.546 [95%CI: 0.523-0.568] respectively. In terms of lipid metabolism, AUC HDL = 0.581 [95%CI: 0.559-0.604] and TG = 0.564 [95%CI: 0.542-0.585]. Besides, 25 (OH) VD AUC = 0.542 [95%CI:0.519-0.564] has the lowest diagnostic efficacy for sarcopenia in our study (**Supplementary Figure 1**). The AUC of multiple factors including fasting insulin, AST/ALT ratio, PA, HDL, FT4, PTC and 25(OH)VD was 0.742 [95%CI: 0.722-0.761], and the combination of fasting insulin with AST/ALT ratio was 0.720 [95%CI: 0.701-0.740] (**Figure 2**).

## Discussion

Sarcopenia is an age-dependent disorder. Skeletal muscle quality and function gradually decline with age, which not only reduces the quality of life of the elderly but also increases the social burden. Due to the different diagnostic criteria and study population, the prevalence of sarcopenia also varies greatly (11). It was reported that, according to the diagnostic criteria developed by the AWGS in 2014, the prevalence of sarcopenia is about 5.5% to 25.7%. The mechanism of sarcopenia not only includes the age, but also nutrition, chronic inflammation, and metabolic derangements. Therefore, we aimed to identify some biomarkers related to nutrition and hormone for the diagnosis of sarcopenia in this study. Patients with sarcopenia had lower value of A/G compared to the control. In our study, we found that patients with sarcopenia had lower values in TP, ALB, A/G, and PA than the healthy (*P*<0.001). Low albumin has been proved to lead to more antioxidant capacities, causing more oxidative damage and therefore muscle breakdown (12, 13). Many elderly people had an inadequate intake of protein due to the decline of digestive and masticatory function, resulting in the imbalance of protein metabolism (14, 15). Malnutrition is frequent in the older and there is an evidence that the supplementation of essential amino acid can prevent the older from sarcopenia (16, 17).

Meanwhile, low 25(OH)VD levels have been confirmed to lead to loss of muscle mass and grip strength through the reduction in II type muscle fiber synthesis identified as fast twitch, which was also consistent with our findings (18, 19). Several studies have already showed that 25(OH)VD could induce muscle cells proliferation, differentiation, and regulate muscle regeneration initiation (20, 21). Besides, Vitamin D could bind to the VDR receptor on muscle fibers and increases their size, improving muscle strength and physical performance. However, the diagnostic performance of 25(OH)VD was the lowest among the biomarkers. The reason may be that the baseline of 25(OH)VD was generally low and 25(OH)VD deficiency is frequent among the older in southwest China. Additionally, we also found that patients had higher levels of PTC. It was found that high levels of PTC could prompt protein degradation through inhibiting mTOR pathway, thus leading to muscle atrophy and affecting the normal physiological function of skeletal muscle (22–25). Patients with sarcopenia had lower levels of fasting insulin than the healthy. The research found that a lack of insulin or insulin resistance leads to accelerated development of sarcopenia. Insulin could mediate accretion of muscle mass through stimulating the mammalian target of rapamycin (mTOR) signaling pathway and promoting the phosphorylation of Akt, thus activating protein synthesis (26). They also showed that physiological hyperinsulinemia increases skeletal muscle protein synthesis (27). Surprisingly, there was a separation between the FT3 and FT4. We found that patients with sarcopenia had lower levels of FT3 but higher levels of FT4 than the healthy. As is known to all, the thyroid hormone plays an important role in regulating the growth and development of our brain and bones. It is also essential for skeletal muscle contractile function and muscle regeneration. Except for regulating the growth, FT3 also regulates skeletal muscle cells by regulating myosin expression (28, 29). Studies have reported that with the increase of age, FT3 will decrease while FT4 will remain stable or increase (30–33). Szlejf et al. revealed that subtle thyroid dysfunction was related to sarcopenia and FT3 had a negative association with muscle mass (29). Although the exact pathogenesis of sarcopenia is still unclear, it is certain that several factors related to age contribute to sarcopenia. Therefore, we inferred that thyroid function may have a certain correlation with the occurrence of sarcopenia, and the specific mechanism needed to be further studied. In the sarcopenia group we found that patients had lower levels of TG and VLDL but higher levels of HDL. Therefore, we inferred that lower TG levels may be a consequence of poor health status associated with sarcopenia (34). A randomized controlled trial revealed that treatment with medium-chain triglycerides could increase the muscle strength and function in elderly with sarcopenia (35). We made multivariate logistic regression analysis and evaluated the predictive performance through the AUC. We found the diagnostic performance of fasting insulin combined with the AST/ALT ratio 0.720 [95%CI: 0.701-0.740] was equal to multiple indicators 0.742 **[**95%CI 0.722-0.761]. From an economic point of view, we strongly recommend fasting insulin and AST/ALT ratio for screening for sarcopenia. Study showed that although non-alcoholic fatty liver disease (NAFLD) was the first cause of liver enzyme abnormalities, reduced ALT has been strongly associated with sarcopenia (36). Besides high serum AST with normal ALT could reflect widespread skeletal muscle pathology (37). Thus, AST to ALT ratio may be a good indicator for frailty and sarcopenia in older patients (38) and multivariate logistic regression model also showed that fasting insulin (OR=0.919, [95%CI: 0.878-0.922] *P*=0.000) and AST to ALT ratio (OR=2.771, [95%CI: 1.544-2.342] *P*=0.000) were risk factors for sarcopenia. Although TP, ALB and PA can all reflect the nutritional status of the body, ALB and PA are more recommended in the evaluation of the nutritional status of patients with sarcopenia, and their corresponding AUC is 0.604 and 0.609, respectively.

## Strengths and Limitations

The current research is a cross-sectional study using baseline data of the WCHAT study and data were collected from four provinces in West China, including Yunnan, Guizhou, Sichuan, and Xinjiang. Although we demonstrated that a significant decrease in triglycerides, albumin, insulin, ALT, vitamin D, and an increase in HDL and AST in sarcopenic patients in the WCHAT study population as prior researches did, in our research we compared the indicators through the ROC curves from a medical laboratory perspective and identified the indictors that were most effective in diagnosing sarcopenia. However, there are also some limitations. Firstly, more data are needed to validate the accuracy of the diagnostic performance. Additionally, only biochemical indicators were included in this research, but other indicators represented immune functions were not available. We will collect new data to validate the accuracy and establish the diagnostic performance of immunological indicators for sarcopenia in future study.

## Data Availability Statement

The raw data supporting the conclusions of this article will be made available by the authors, without undue reservation.

## Author Contributions

MY: Conceptualization, Data curation, Formal analysis, Writing - original draft, Writing - review and editing. HZ: Data curation, Writing - review and editing. LH: Data curation, Writing - review and editing. YD: Data curation, Writing - review and editing. HW: Data curation, Writing - review and editing. QL and FD: Funding acquisition, Data curation, Writing - review and editing. JY and YH: Conceptualization, Writing - review and editing. All authors contributed to the article and approved the submitted version.

## Funding

This research is supported by Grant National Key R&D Program of China (2020YFC2005600 and 2020YFC2005603), Sichuan Science and Technology Agency of Sichuan Province (2020YFS0185) and Sichuan Science and Technology Program (2019YFS0277).

## Conflict of Interest

The authors declare that the research was conducted in the absence of any commercial or financial relationships that could be construed as a potential conflict of interest.

## Publisher’s Note

All claims expressed in this article are solely those of the authors and do not necessarily represent those of their affiliated organizations, or those of the publisher, the editors and the reviewers. Any product that may be evaluated in this article, or claim that may be made by its manufacturer, is not guaranteed or endorsed by the publisher.
