# Corrigendum: Diagnostic Performance of Clinical Laboratory Indicators With Sarcopenia: Results From the West China Health and Aging Trend Study

**DOI:** 10.3389/fendo.2022.852295

**Published:** 2022-02-01

**Authors:** Mengting Yin, He Zhang, Qianhui Liu, Fei Ding, Yiping Deng, Lisha Hou, Hui Wang, Jirong Yue, Yong He

**Affiliations:** ^1^ Department of Laboratory Medicine, West China Hospital, Sichuan University, Chengdu, China; ^2^ Department of Geriatrics and National Clinical Research Center for Geriatrics, West China Hospital, Sichuan University, Chengdu, China

**Keywords:** sarcopenia, AST/ALT ratio, fasting insulin, diagnostic performance, metabolism

In the article as originally published, the author Hui Wang’s affiliation was incorrectly indicated as affiliation 1, Department of Laboratory Medicine, West China Hospital, Sichuan University, Chengdu, China. This author’s correct affiliation is affiliation 2, Department of Geriatrics and National Clinical Research Center for Geriatrics, West China Hospital, Sichuan University, Chengdu, China.

The authors apologize for this error and state that this does not change the scientific conclusions of the article in any way. The original article has been updated.

## Publisher’s Note

All claims expressed in this article are solely those of the authors and do not necessarily represent those of their affiliated organizations, or those of the publisher, the editors and the reviewers. Any product that may be evaluated in this article, or claim that may be made by its manufacturer, is not guaranteed or endorsed by the publisher.

