# Supplementary material for: Diagnostic Performance of Clinical Laboratory Indicators With Sarcopenia: Results From the West China Health and Aging Trend Study
Source: Front Endocrinol (Lausanne). 2021 Dec 10;12:785045. doi: 10.3389/fendo.2021.785045 (PMC8702640; doi:10.3389/fendo.2021.785045)
Supplement: Supplementary file 1 [file DataSheet_1.docx]

Supplementary Table 1

|  |  | sarcopenia |  | control |  | *P* value |
| --- | --- | --- | --- | --- | --- | --- |
| DBIL  TBIL |  | 5.96±3.57  18.41±7.65 |  | 5.90±2.69  19.09±7.74 |  | 0.310  0.004 |
| IDBIL |  | 12.45±4.93 |  | 13.20±5.45 |  | 0.001 |
| CREA |  | 80.17±18.17 |  | 80.21±19.13 |  | 0.134 |
| UA |  | 320.30±86.22 |  | 329.40±82.08 |  | 0.001 |
| 0-GLU |  | 5.47±1.96 |  | 5.57±1.66 |  | 0.000 |
| WBC |  | 5.96±1.97 |  | 5.84±1.58 |  | 0.245 |
| RBC |  | 4.83±0.58 |  | 4.96±0.58 |  | 0.000 |
| HCT |  | 46.58±5.76 |  | 47.36±5.63 |  | 0.001 |
| MCV |  | 96.20±5.94 |  | 95.34±5.68 |  | 0.000 |
| MCH |  | 30.40±2.08 |  | 30.18±1.99 |  | 0.001 |
| MCHC |  | 316.20±10.29 |  | 316.14±8.79 |  | 0.909 |
| PLT |  | 170.00±57.67 |  | 170.30±55.81 |  | 0.963 |
| PCT |  | 0.18±0.05 |  | 0.18±0.05 |  | 0.096 |
| UREA |  | 5.46±1.70 |  | 5.37±1.59 |  | 0.229 |
| LYMF |  | 1.73±0.76 |  | 1.78±0.56 |  | 0.003 |
| MPV |  | 10.95±1.58 |  | 11.12±1.53 |  | 0.003 |
| PDW |  | 13.58±2.22 |  | 13.84±2.19 |  | 0.001 |
| GLO |  | 27.93±4.16 |  | 27.61±5.01 |  | 0.047 |
| CHOL |  | 4.76±0.98 |  | 4.79±0.90 |  | 0.301 |
| GPR |  | 62.31±9.33 |  | 60.91±8.45 |  | 0.000 |
| LPR |  | 30.53±8.57 |  | 31.70±7.80 |  | 0.000 |
| MPR |  | 7.16±1.48 |  | 7.38±1.47 |  | 0.000 |
| GRAN |  | 3.74±1.53 |  | 3.58±1.33 |  | 0.015 |
| RDWA |  | 53.34±4.77 |  | 51.99±4.40 |  | 0.000 |
| MID |  | 0.48±0.38 |  | 0.47±0.15 |  | 0.361 |
| PDWR |  | 14.89±0.93 |  | 14.63±0.76 |  | 0.000 |
| P-LCR |  | 33.62±10.70 |  | 34.69±10.37 |  | 0.003 |

**Note:** DBIL, direct bilirubin; IBIL, indirect bilirubin; TBIL, total bilirubin; CREA, creatinine; UA, uric acid; 0-GLU, fasting glucose; WBC, white blood cell; RBC, red blood cell; HCT, hematocrit; MCV, mean corpuscular volume; MCVC, mean corpuscular hemoglobin concentration; PLT, platelet; PCT, procalcitonin; LYMF, Percentage of lymphocytes; MPV, mean platelet volume; PDW, platelet distribution width; GLO, globulin; CHOL, cholesterol; GPR, gamma glutamyl transpeptidase platelet ratio; LPR, percentage of lymphocytes; MPR, median percentage; GRAN, neutrophil granulocyte percentage; RDWA, red cell distribution width; MID, percentage of intermediate cells; PDW, platelet distribution width; P-LCR, platelet-large cell ratio.


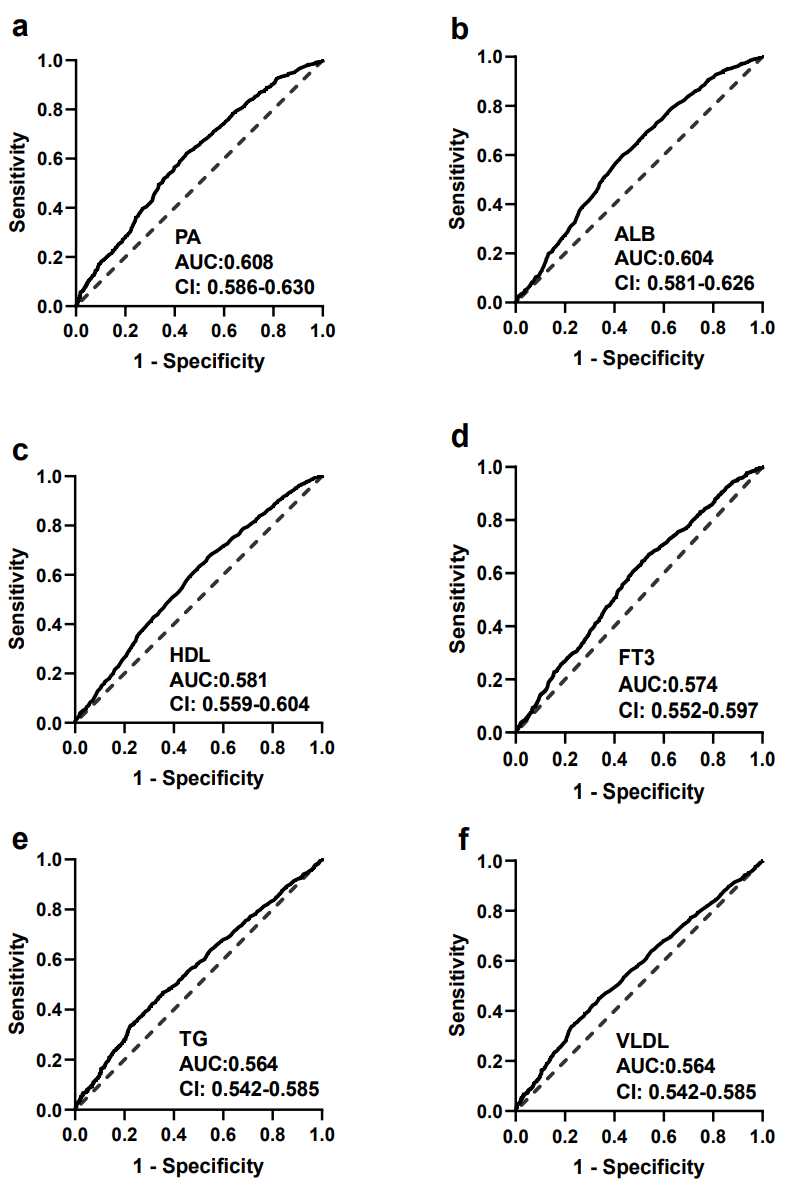


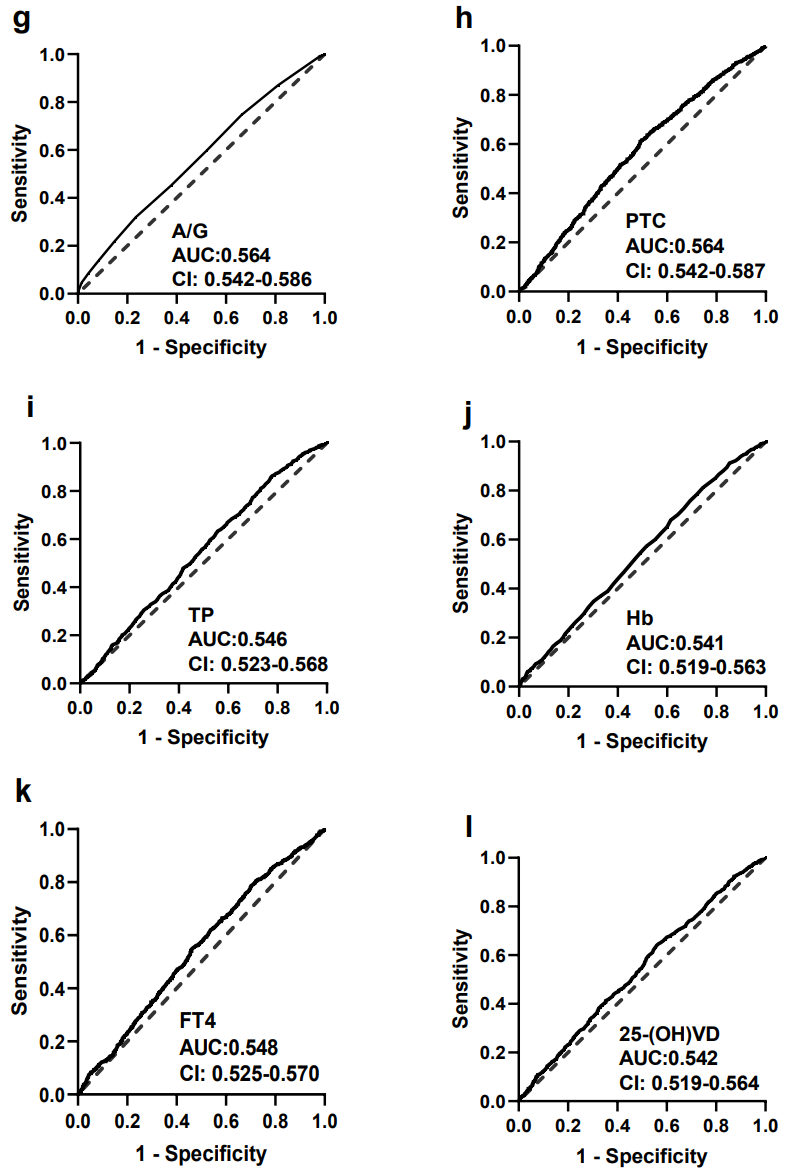


**Fig 1** The predictive performance of indicators for sarcopenia. a PA, AUC = 0.609 [95%CI: 0.586-0.630], b ALB, AUC = 0.604 (95%CI: 0.581-0.626), c HDL, AUC = 0.581 [95%CI: 0.560-0.604], d FT3, AUC = 0.573 [95%CI: 0.552-0.597], e TG, AUC = 0.564 [95%CI: 0.542-0.585], f VLDL, AUC = 0.569 [95%CI: 0.547-0.590], g ALB, AUC = 0.604 [95%CI: 0.581-0.626], h PTC, AUC = 0.561 (95%CI: 0.539-0.584), i TP, AUC = 0.546 [95%CI: 0.523-0.568], j Hb, AUC = 0.546 [95%CI: 0.523-0.569], k FT4, AUC = 0.548 [95%CI: 0.525-0.570], l 25(OH)VD, AUC = 0.542 [95%CI: 0.519-0.564]. Note: PA, prealbumin; FT3, free triiodothyronine; FT4, free tetraiodothyronine; ALB, albumin; ALT, alanine aminotransferase; AST, aspartate aminotransferase; TP, total protein; TG, triglycerides (TG); HDL, high-density lipoprotein; VLDL, very low-density lipoprotein; PTC, total cortisol; Hb, hemoglobin.
